# Are “Theory of Mind” Skills in People with Epilepsy Related to How Stigmatised They Feel? An Exploratory Study

**DOI:** 10.1155/2016/5025174

**Published:** 2016-08-18

**Authors:** A. J. Noble, A. Robinson, A. G. Marson

**Affiliations:** ^1^Department of Psychological Sciences, Institute of Psychology, Health and Society, University of Liverpool, The Whelan Building, Liverpool L69 3GL, UK; ^2^Department of Molecular and Clinical Pharmacology, Institute of Translational Medicine, University of Liverpool, Liverpool L69 3GL, UK

## Abstract

Feelings of stigma are one of the main burdens reported by people with epilepsy (PWE). Adults with temporal or frontal lobe epilepsy and children with idiopathic generalised epilepsy are at risk of Theory of Mind (ToM) deficits. ToM refers to social cognitive skills, including the ability to understand the thoughts, intentions, beliefs, and emotions of others. It has been proffered that ToM deficits may contribute to the feelings of stigma experienced by PWE. In this study we tested this for the first time. We also determined the association between clinical and demographic factors and ToM performance. Five hundred and three PWE were recruited via epilepsy organisations and completed measures online. Feelings of stigma were measured using Jacoby's Stigma Scale, whilst the Reading the Mind in the Eyes Test and the Faux Pas Test measured ToM. The median age of participants was 37 years, their median years living with epilepsy were 15, and 70% had experienced seizures in the prior 12 months. Feelings of stigma held a negligible, negative, and nonsignificant association with ToM performance (*r*_*s*_  −0.02 and −0.05). Our results indicate that the ToM model for understanding epilepsy stigma has limited utility and alternative approaches to understanding and addressing epilepsy-related stigma are required.

## 1. Introduction

Stigma continues to be one of the main burdens reported by people with epilepsy (PWE) [1–3]. Goffman [4] defined stigma as referring to the loss of status that can arise from being in possession of an attribute, in this case epilepsy that has been culturally defined as “undesirably different” and means those in possession of it are seen as “not quite human.” In UK, 14–50% of PWE feel stigmatised [5–7], with those with more recent diagnoses or ongoing seizures being most likely to experience such feelings [6, 7].

Feelings of stigma have important implications for PWE. Qualitative studies suggest they contribute towards the “hidden distress” of the condition, with patients describing how they can feel ashamed and guilty about their diagnosis and afraid to disclose it [8–10]. Fifty percent of children identify stigma as the worst part of having epilepsy [11]. In adult patients, feelings of stigma have been found to be associated with increased depression and anxiety, impaired physical health, reduced self-esteem, low life-satisfaction, and medication nonadherence [12].

The history of epilepsy shows the condition that has attracted many wayward theories about its cause and treatment [13]. When considering how to ameliorate epilepsy-related stigma, the orthodox view has been that the general public often continues to be ignorant about epilepsy [10, 14]. Much attention has therefore been given to improving the knowledge and attitudes of the public. Unfortunately, effective interventions are still lacking and stigma continues [15, 16]. It is important therefore to still consider alternative models for understanding epilepsy-related stigma.

One such model has recently emerged. Specifically, adults with seizures arising from the temporal or frontal lobes and children with idiopathic generalised epilepsy demonstrate large deficits in the high-level social cognitive skills referred to as “Theory of Mind” (ToM) [17, 18]. It has been suggested that these could account for some of the psychobehavioral difficulties experienced by PWE [19–25], with stigma being noted by some as one of the difficulties.

ToM refers to the complex set of skills that include the ability to understand the thoughts, intentions, beliefs, and emotions of others [26]. As outlined by Stewart et al. [18], such skills differ from basic social perception abilities in that they relate to the detection of ambiguous or covert social cues in order to understand both cognitive and affective internal mental states (e.g., eye-gaze expression; irony).

The presumed logic for the role that ToM has been suggested to have for stigma is that ToM deficits could hamper a patient's ability to negotiate social interactions, including accurately interpreting and responding to the actions of other people, and this could contribute to mistaken feelings of stigmatisation. Such deficits could in this sense potentially explain why PWE have often been found to* feel* stigmatised, despite struggling to recount episodes of actual discrimination [5, 8, 27].

ToM deficits in epilepsy may result from a disruption of the prefrontal, orbitofrontal, mesolimbic, and anterior and posterior temporolateral brain structures which appear to support ToM [28–30]. They may also be related to degraded developmental acquisition due to children and adolescents with epilepsy having fewer opportunities to develop such skills [21].

To date, there has been no formal examination of the relationship between ToM deficits and feelings of stigma in epilepsy. There are reasons though, why the model warrants testing. Firstly, in other conditions, such as schizophrenia and paranoia, ToM mechanisms have been implicated in the pathogenesis of psychiatric symptoms and altered personality and behavior [31–33]. Moreover, there is recent evidence to suggest ToM impairments may be associated with some other psychobehavioral difficulties reported by PWE. Wang et al. [22] found ToM scores were associated with scores on a Taiwanese measure of social functioning; Giovagnoli et al. [19] found a link between ToM performance and quality of life perceptions.

The second reason is that should ToM deficits be associated with stigma; it would have meaningful treatment implications since it would suggest a different approach to addressing stigma [34]. Finally, the ToM model is potentially a contentious one. It does not refute the health consequences of felt stigma. However, in indicating that the cause of some feelings of stigma might be cognitive, it could be seen by some that the model is implying epilepsy stigma which is less “real.” Evidence on its actual scientific basis is therefore required.

We therefore conducted an exploratory study to obtain initial evidence on the magnitude of association between the ToM skills in PWE and their feelings of stigma. We also explored what patient-related factors were associated with ToM performance.

## 2. Methods

### 2.1. Participants

Participants were PWE affiliated with epilepsy patient organisations or interest groups in the UK and Republic of Ireland (see Acknowledgments). All were aged ≥18 years and reported a clinical diagnosis of epilepsy. People were excluded if they could not provide informed consent or independently complete questionnaires in English.

### 2.2. Procedure

Data were collected as part of a wider, online cross-sectional project. The purpose of that study was to understand the preferences of PWE and their significant others when it comes to talking about and labelling epilepsy. As part of that study, participants completed an online questionnaire pack. Of relevance to the current report, patients were asked about their feelings of stigma and to complete two ToM tests.

With regard to recruitment, an advertisement was sent by the epilepsy organisations between December 2015 and February 2016 to those on their mailing lists and adverts placed in their newsletters. Persons wanting to take part were directed to an online study page hosted by Qualtrics.

The University of Liverpool's Institute of Psychology, Health and Society Research Ethics Committee approved the study (IPHS-1516-SMc-105) and informed consent was obtained from all participants.

### 2.3. Measures

#### 2.3.1. Felt Stigma

Jacoby's [5, 7] Stigma of Epilepsy Scale, measured the extent to which participants felt stigmatised by epilepsy. This established measure is both reliable and valid (*α* coefficients = 0.77 to 0.85) [6, 7, 35]. It asks individuals to what extent, because of their epilepsy, they feel other people (1) are uncomfortable with them, (2) treat them as inferior, and (3) prefer to avoid them. Participants respond to each statement using a 4-point Likert-type scale. Scores range from 0 to 9. A score of 0 indicates the person does not feel stigmatised, score of 1–6 indicates the mild to moderate stigma, and scores of 7–9 indicate severe stigma.

#### 2.3.2. ToM Skills

Two advanced ToM tests, appropriate for use with adults, were used.

*(1) Faux Pas Task-Short Version (FPT) [21, 36].* It uses three stories (13, 15, and 16 from full version) to estimate a participant's ability to recognize and understand social Faux Pas. After being presented with each story, the participant is asked four questions. One control question checks the participant has understood the story. The remaining three questions ask about interpersonal relations and emotional states. Correct answers require that (a) the subject can understand the Faux Pas correctly; (b) he or she can correctly impute the mental state of another; and (c) he or she can attribute emotions to another. One point is awarded for each test question answered correctly.

Total scores range from 0 to 9, with higher scores indicating better ToM performance. To minimize load on memory, each story remained on the screen whilst the questions were asked. The FPT [37] is the most commonly used measure to assess for ToM skills in epilepsy [17] and the short version has been used in prior epilepsy studies since it reduces participant burden [20, 21, 38] and because reliability analysis between it and the full version has revealed a sufficient correlation (*r* = 0.74) [21]. 

*(2) Reading the Mind in the Eyes Test-Short Version (RMET) [20, 39].* The RMET asks participants to choose which of four presented words best describes the mental or affective state of a person whose eyes are shown in a picture (e.g., terrified, amused, regretful, and flirtatious). In doing this, the RMET test aims to measure higher-level facial emotion perception.

The short version includes 10 pictures being presented (item numbers 5, 6, 9, 11, 16, 19, 23, 26, 30, and 36). Reliability analysis between the long and short version has revealed a sufficient correlation between the two when used with PWE (*r* = 0.78) [20]. Total scores range from 0 to 10, with higher scores indicating better performance. The test makers recommend that a glossary of all words is available to participants whilst they are viewing the pictures and that any participants scoring zero should be removed as this likely reflects an overall difficulty in understanding test instructions.

#### 2.3.3. Covariate Measures

To allow us to fully describe the sample and explore what factors were associated with ToM performance, participants reported their demographics and medical history. This included age of epilepsy onset, first spoken language, number of antiepileptic drugs currently prescribed, and seizure frequency. The latter was captured using Thapar et al.'s [40] scale which asks about the number of seizures (of any type) the patient experienced in the previous 12 months. Using the Impact of Epilepsy scale (Revised) [41], participants also rated the extent to which epilepsy imposed restrictions on their life. Finally, ToM performance may hold an association with verbal intelligence. As such, an estimate of each participant's verbal intelligence was obtained by asking them to complete the Spot-the-Word test [42].

### 2.4. Statistics

The primary objective was to estimate the association between patients' reports of felt stigma and performance on the two ToM tests. Whilst the ToM model for stigma suggests ToM skills should be inversely related to felt stigma, there was no evidence on the likely magnitude of the association so as to inform a sample size calculation. Therefore, given the diverse sample and that not all patients would likely demonstrate ToM deficits, we adopted the conservative approach of assuming that ToM scores might account for 10% of the variance in patients' stigma scores. Using formulae provided by Algina and Olejnik [43], a sample of 493 participants with complete data was deemed to be required to allow us to estimate such an association with a high degree of precision (i.e., ±0.5%) and confidence (i.e., 95%).

Descriptive statistics were used to examine participants' characteristics. Box plots and scatter-plots helped to visualise how performance on the ToM tests related to the degree of stigma participants reported. Due to the ordinal nature of the stigma scale, the bivariate association between performances on the two ToM tests and scores on the stigma scale were examined using Spearman's rank-order correlation test (*r*_*s*_, along with 95% confidence intervals, CI). As a secondary analysis we computed the association between ToM test performances and stigma separately for those who were seizure-free and those with ongoing seizures.

To explore which factors were associated with ToM performance, scores on the measures were treated as continuous and linear regression, with robust standard errors, was used. Any variables significantly associated (*P* < 0.05) with a ToM performance were then simultaneously entered into multiple regression analyses to identify parsimonious predictors. Unstandardized coefficients (*β*), along with 95% CIs, and *R* ^2^ are presented.

Analyses were completed using Stata 11 (Stata Corporation, College Station, TX, USA) and StatsDirect 2.7.8 (StatsDirect Ltd., Cheshire, United Kingdom).

## 3. Results

### 3.1. Participants

A total of 589 PWE were recruited, of which 503 (85.4%) had complete data and were included in the analyses. Those with missing data did not differ significantly from those with patients without missing data in their current age, age of epilepsy onset, sex, seizure frequency, or total stigma score. Those with missing data typically had incomplete data across all measures that they were asked to do. It was not restricted to ToM tests or another measure.

### 3.2. Clinical and Demographic Characteristics

Of the 503 participants, the majority were female (78.9%) (Table 1). The median age of participants was 37 years (IQR = 27–47, range: 18–79). Two-thirds (66.0%) reported no known cause for their epilepsy and 15.3% epilepsy resulting from an acquired brain injury. Only 25.6% of participants reported having been seizure-free in the prior 12 months. Most (74.6%) reported that their seizure type included generalised convulsive seizures. Educational attainment was high, with 74% of participants having achieved a postschool qualification (e.g., higher school-leaving certificate (A-level, degree)). Nearly all participants identified themselves as being White British (94.8) and English (98.2%) as their first language.

### 3.3. Felt Stigma, ToM Scores, and Association between Them

The median score on stigma scale was 3 (IQR = 1–6; range 0–9), with most (80.5%) participants in the sample reporting at least some feelings of stigma due to their epilepsy. Most (61.2%) reported mild-moderate stigma and a minority (19.3%) severe stigma.

The mean score for the RMET was 6.93 (SD = 1.67), with no subjects scoring 0. The mean score on the FPT was 5.48 (SD = 2.20). Only minority of participants responded incorrectly to the questions used to check comprehension for the stories (9 (1.8%) for story 1, 38 (7.6%) for story 15, and 58 (11.5%) for story 16). When those who failed any comprehension questions were excluded (89; 17.6%), the mean score on the FPT was 5.84 (SD = 2.01). The total scores for the two ToM tests held a small, significant positive correlation with one another (*r* = 0.170, *P* < 0.001).

**Figure 1:**
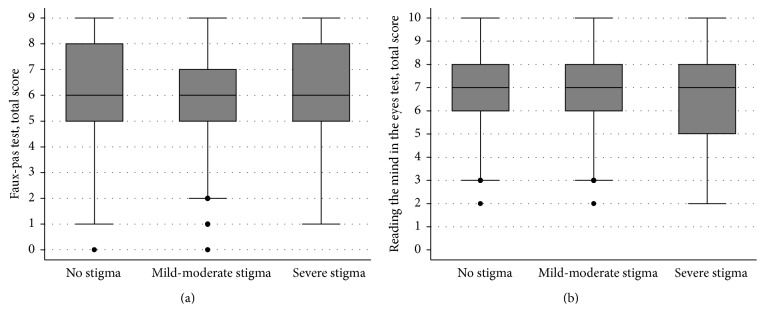
Box plots representing the score profile for participants on the Theory of Mind tests according to the extent of felt stigma they reported. (a) shows scores on the Faux Pas Test; (b) shows scores on the Reading the Mind in the Eyes test. Notes: dark horizontal lines represent the median, with the boxes representing the 25th and 75th percentiles, the whiskers the 5th and 95th percentiles, and outliers represented by dots. For the Faux Pas Test, the data relates to only those participants who did not fail any comprehension control questions.

Stigma and ToM performance were found to be largely independent constructs (Figure 1). Correlational analyses showed that the two factors shared little covariance. Increased felt stigma held only a negligible, negative, and nonsignificant association with performance on the RMET (*r*_*s*_ = −0.02, 95% CI −0.11, 0.05) and FPT (*r*_*s*_ = −0.05, 95%  −0.14, 0.03). The relationship between ToM test performances and stigma remained small and nonsignificant when computed separately for those with and without ongoing seizures (Table 2).

### 3.4. Clinical and Demographic Factors Associated with ToM Scores

For the RMET, only duration of epilepsy and performance on the “Spot-the-Word” were associated with the total test score, with longer duration (*β* = −0.015, 95% CI −0.025, −0.004) and worse performance on the verbal IQ test (*β* = 0.081, 95% CI 0.053, 0.109) being associated with a worse RMET score (Table 3). The final model based on these two factors could, however, account for only 8% variance in performance on the RMET. For the FPT, a number of significant associations were identified by univariate screening and entered into multiple regression models. In the adjusted analyses, the variables which remained significantly associated with worse performance were being unemployed (*β* = −0.529, 95% CI −1.006, −0.051) and poorer performance on the verbal IQ measure (*β* = 0.081, 95% CI 0.044, 0.118). For the FPT, the final model accounted for 6% of variance in scores on the FPT.

## 4. Discussion

### 4.1. Main Findings

The clinical significance of Theory of Mind (ToM) deficits in epilepsy are starting to be explored. This study for the first time examined the relationship between the ToM skills of people with epilepsy (PWE) and their perceptions of stigma. In this exploratory study, we found that ToM skills and felt stigma were largely unrelated. Patients' scores on the ToM tests shared less than 1% of variance with the patients' concurrent feelings of stigma.

Strengths of our study include its large sample. Over 500 PWE completed the ToM tests and stigma scale. Such a large sample means the estimates we provide have narrow confidence intervals. The fact that we did not find stigma to be related to ToM aligns with the recent failure of studies to find a consistent link between ToM and affective states in epilepsy, such as depression and anxiety [19, 20, 25].

Using metaregression Stewart et al. [18] recently explored factors associated with ToM performance in epilepsy. They identified that the magnitude of ToM deficits increased in patients with temporal (but not frontal) lobe epilepsy as participants' age decreased. As the number of studies on ToM remains relatively small, Stewart et al. could only include 12 studies in their review which together included 595 PWE. To further develop the evidence base, we explored what factors were associated with ToM skills in our sample of over 500 PWE. For the RMET, adjusted analyses identified increased duration of epilepsy and lower scores on the Spot-the-Word test were significantly associated with lower ToM skills. For the FPT, having had a seizure in the prior 12 months, being unemployed, and lower scores on the Spot-the-Word test were significantly associated with lower ToM skills. Our cross-sectional design means that conclusions cannot be made about the direction of the relationship between these. However, that test of verbal intelligence was consistently associated with ToM performance, does mirror what has been found in the wider literature [44], and underlines the importance for studies to control for intelligence when comparing ToM performance between groups.

### 4.2. Potential Study Limitations

In forwarding evidence that refutes the utility of the ToM model, it is important to consider alternative reasons why our study might not have found evidence to support the model.

Perhaps most importantly, recruitment was here not restricted to just people whose type of epilepsy is known to be related to large ToM deficits, namely, adults with temporal or frontal lobe epilepsy and children with idiopathic generalised epilepsy [18]. Rather, persons with all types of epilepsy could take part. This was because our study was nested within a wider project and participant inclusion/exclusion criteria resulted from this. The online nature of recruitment also meant insufficient information was available on participants' type of epilepsy so as to allow us to select for inclusion only those with one of the aforementioned epilepsies. By including people in our sample who might not have been at risk of ToM deficits, but who could have still experienced stigma, could have limited the opportunity for us to detect a sizeable correlation between ToM and stigma. A close examination of the characteristics of our sample does not though support this explanation for our result.

Specifically, persons with uncontrolled epilepsy were overly represented amongst our sample. Seventy percent of our participants had seizures in the prior 12 months compared to the 48% one finds in the wider UK epilepsy population [45]. This is important as such persons typically have a localization-related epilepsy [46] and ~90% of them have either temporal or frontal lobe epilepsy [47]. It seems likely therefore that people at risk of ToM deficits did ultimately comprise a sizeable proportion of our sample.

A comparison of our participants' scores on the ToM tests to those of participants in other studies supports this assertion. It shows that ToM deficits were common in our sample. Schacher et al. [21] used the same FPT test as we did with patients with epilepsy from mesial temporal lobe sclerosis, patients with epilepsy outside of the temporal and frontal lobes and healthy controls. With a mean score of ~6.3, the first group demonstrated significantly worse ToM skills compared to those with other types of epilepsy (mean ~7.5) and the healthy controls (mean ~8.5). Our participants' mean score on the same FPT was worse than all of these groups, even after excluding those participants who demonstrated comprehension difficulties (mean 5.84).

To further rule out the possibility that the small association we found was an artefact of our recruitment strategy, we reported on the association between ToM performance and stigma separately for those participants with and without ongoing seizures. As stated above, those with ongoing seizures are more likely to have temporal or frontal lobe epilepsy and so be at most risk of ToM deficits. However, we did not find that the association changed in any meaningful way. It remained both small and nonsignificant in both subgroups.

There is no consensus regarding the best way to assess ToM in PWE. Meta-analyses have found deficits are fairly robust and occur across different tests [18]. We used two advanced tests to minimize ceiling effects. However, it was necessary to use shortened versions of the tasks to limit participant burden. These versions have been found to be highly correlated with the full versions in prior studies [20, 21]. A consequence of their use though is that score ranges are restricted. This could have limited the possible size of correlation between ToM and stigma scores. Future studies would therefore be advised to use the full version of the tests and potentially others to ensure that the estimate our study provides is robust. Having said this, it seems reasonable to still contend that the relationship between ToM and stigma is not profound. To account for even 10% of the variance in stigma scores, the correlation between ToM performance and stigma would need to be six times larger than that found by our study.

### 4.3. Future Directions

Our patients were younger [45] and more educated [48] than those in the wider epilepsy population. Minority ethnic groups were also underrepresented [49]. This likely occurred because we restricted participation to patients who had internet access and who were affiliated with epilepsy organisations. Whilst 86% of UK households have internet access [50], cost is a barrier as is older age. Most (80%) of our participants also reported feeling stigmatised (albeit at a mild level). Previous studies found only 52% of those associated with epilepsy organisations report stigma [51]. This may be because we used the more sensitive, revised version of the Jacoby Stigma Scale [5, 7]. Nevertheless, this and the other features of our sample mean that it is important for future studies to test how well our findings generalise to more representative samples.

Since ToM does not appear to offer a reasonable explanation for felt stigma in epilepsy it is important to consider where research attention would best now be focused. One reason that the ToM model seemed attractive was that it offered an explanation for why PWE can report high felt stigma, despite low rates of explicit discrimination. Considering other explanations for this phenomenon offer some direction for research. One is that a disconnect occurs because perceptions of stigma result from both experiences of enacted stigma, but also from the anticipation of future negative experience. Link [52] suggested that individuals with stigmatising conditions assume from prevailing illness stereotypes that they will be devalued and discriminated against. This can lead them to adopt coping strategies, like secrecy and social withdrawal, which reinforce feelings of stigma.

As outlined by Jacoby et al. [3], what may also explain the disconnect is that efforts to document enacted stigma typically focus on episodes at the “hard end,” such as being dismissed from work because of one's diagnosis, and ignore more subtle expressions (e.g., difficulties obtaining insurance [53]).

The implications of both explanations is that, in order to attenuate stigma, efforts need to continue to be made to improve societal attitudes towards epilepsy and to address important knowledge gaps towards epilepsy that many have. Unfortunately, there continues to be a shortage of empirically evaluated and scalable interventions that can be used to do this [54]. There is a need therefore for continued investment in this area.

## 5. Conclusion

To reduce epilepsy-related stigma, a clear understanding of the mechanisms that bring it about is required. In this study we tested for the first time suggestions that some perceptions of stigma might be related to deficits on behalf of PWE in high-order social cognitive skills referred to as Theory of Mind. Our exploratory study found little evidence to support such a model. Alternative approaches to understanding and addressing epilepsy-related stigma are therefore required.

## Figures and Tables

**Table 1:** Clinical and demographic characteristics of participants.

Factors	All participants
*Age (years)*	
Median (IQR)	37.0 (27.0, 47.0)
<50	401 (79.7)
≥50	102 (20.3)

*Sex (n/%)*	
Male	106 (21.1)
Female	397 (78.9)

*Highest educational attainment (n/%)*	
Basic school certificate or lower	131 (26.0)
Advanced school certificate or equivalent	105 (20.9)
University degree, diploma, or higher	267 (53.1)

*Employment (n/%)*	
Employed (full/part-time)/student	303 (60.2)
Homemaker/others	97 (19.3)
Unemployed	103 (20.5)

*Epilepsy onset*	
Median (IQR)	18.0 (12.0, 30.0)
≤12 years	142 (28.2)
>12 years	361 (71.8)

*Marital status*	
Single	241 (47.9)
Married	217 (43.1)
Divorced	45 (8.9)

*Duration of epilepsy*	
Median (IQR)	15 (6, 27)

*Antiepileptic medication (n/%)*	
Monotherapy or none	175 (34.8)
Polytherapy	328 (65.2)

*Seizures (any type) prior 12 months* ^a^	
Median (IQR)	5 (0, 10)
No	151 (30.0)
Yes	352 (70.0)

*Experience convulsive seizures? (n/%)*	
Yes	374 (74.4)
No	129 (25.6)

*Reported cause of epilepsy*	
Unknown	332 (66.0)
Acquired brain injury	77 (15.3)
Others	94 (18.7)

*Impact of epilepsy *	
Median (IQR)	−9 (14.0, −4.0)

*Theory of mind test (mean, SD)*	
Mind in eyes test (higher = better ToM)	6.93 (1.67)
Faux pas test (higher = better ToM)	5.84 (2.01)

*Notes*: IQR: interquartile range; *n*: number; SD: standard deviation; ^a^Thapar et al. [40] scale which asks “How many attacks have you had in the last 12 months?” The patient can choose from the following ordinal categories: 0, 1, 2, 3, 4, 5, 6, 7, 8, 9, 10, or more.

**Table 2:** Association between Theory of Mind test performance and stigma score according to participant's seizure status.

Theory of Mind test	Seizure-free *n* = 151		Ongoing seizures *n* = 352	
	M (SD)	*r*_*s*_ (95% CI)	M (SD)	*r*_*s*_ (95% CI)
Mind in eyes test (higher = better ToM)	7.02 (1.76)	−0.01 (−0.12, 0.08)	6.89 (1.64)	−0.01 (−0.17, 0.14)
Faux pas test (higher = better ToM)	5.82 (2.04)	−0.01 (−0.16, 0.15)	5.34 (2.25)	−0.03 (−0.14, 0.06)

*Notes*: CI: confidence interval; *n*: number; *r*_*s*_: Spearman's rank-order correlation test; SD: standard deviation; ToM: Theory of Mind.

**Table 3:** Association between demographic/clinical factors and Theory of Mind test performance.

Factors	Theory of Mind test					
	Reading mind in the eyes test			Faux pas test		
	Mean (SD)	Unadjusted B (95% CI)	Adjusted B (95% CI)	Mean (SD)	Unadjusted B (95% CI)	Adjusted B (95% CI)
*Age (years)*	—	−0.005 (−0.017, 0.006)	—	—	−0.008 (−0.022, 0.006)	—

*Sex (n/%)*						
Male	6.90 (1.79)	1.00 Reference		9.83 (3.76)	1.00 Reference	
Female	6.93 (1.64)	0.033 (−0.344, 0.412)	—	10.42 (3.51)	0.437 (−0.050, 0.926)	—

*Highest educational attainment (n/%)*						
Basic school certificate or lower	6.58 (1.76)	1.00 Reference		9.91 (3.60)	1.00 Reference	
Advanced school certificate or equivalent	7.04 (1.72)	0.145 (−0.222, 0.513)	—	10.03 (3.62)	−0.182 (−0.657, 0.293)	—
University degree, diploma, or higher	7.05 (1.59)	0.271 (−0.024, 0.567)	—	10.59 (3.52)	**0.438 (0.053, 0.824)**	0.096 (−0.299, 0.492)

*Employment (n/%)*						
Employed (full/part-time)/student	7.06 (1.60)	1.00 Reference		10.63 (3.48)	1.00 Reference	
Homemaker/others	6.63 (1.77)	−0.363 (−0.751, 0.024)	—	10.03 (3.76)	−0.233 (−0.723, 0.257)	—
Unemployed	6.80 (1.78)	−0.159 (−0.539, 0.221)	—	9.59 (3.55)	**−0.661 (−1.135, −0.186)**	**−0.529 (−1.006, −0.051)**

*Epilepsy onset*						
≤12 years	6.69 (1.89)	1.00 Reference		5.27 (2.20)	1.00 Reference	
>12 years	7.00 (1.60)	0.316 (−0.059, 0.691)	—	5.55 (2.20)	0.278 (−0.174, 0.731)	—

*Duration of epilepsy*	—	**−0.011 (−0.021, −0.001)**	**−0.015 (−0.025, −0.004)**	—	−0.003 (−0.017, 0.010)	—

*Marital status*						
Single	6.85 (1.72)	1.00 Reference		10.31 (3.55)	1.00 Reference	
Married	6.99 (1.61)	0.110 (−0.183, 0.405)	—	10.20 (3.63)	−0.094 (−0.485, 0.295)	—
Divorced	7.06 (1.75)	0.147 (−0.383, 0.678)	—	10.71 (3.40)	0.172 (−0.515, 0.861)	—

*Antiepileptic medication (n/%)*						
Monotherapy or none	7.04 (1.66)	1.00 Reference		10.38 (3.55)	1.00 Reference	
Polytherapy	6.82 (1.69)	−0.225 (−0.519, 0.068)	—	10.22 (3.59)	−0.092 (−0.478, 0.293)	—

*Seizures (any type) prior 12 months* ^a^ * (n/%)*						
No	7.02 (1.76)	1.00 Reference		5.82 (2.04)	1.00 Reference	
Yes	6.89 (1.64)	−0.134 (−0.464, 0.195)	—	5.34 (2.25)	**−0.477 (−0.880, −0.074)**	−0.304 (−0.708, 0.099)

*Experience convulsive seizures? (n/%)*						
Yes	6.94 (1.68)	1.00 Reference		10.30 (3.52)	1.00 Reference	
No	6.89 (1.66)	−0.044 (−0.379, 0.290)	—	10.31 (3.71)	−0.102 (−0.554, 0.349)	—

*Reported cause of epilepsy*						
Unknown	6.98 (1.66)	1.00 Reference		10.41 (3.47)	1.00 Reference	
Acquired brain injury	7.01 (1.57)	0.095 (−0.291, 0.482)	—	10.03 (4.12)	−0.176 (−0.771, 0.418)	—
Others	6.69 (1.79)	−0.296 (−0.692, 0.100)	—	10.13 (3.43)	−0.298 (−0.773, 0.176)	—

*Impact of epilepsy *	—	0.001 (−0.020, 0.024)	—	—	0.008 (−0.020, 0.038)	—

*Spot the word test (corrects)*	—	**0.076 (0.048, 0.104)**	**0.081 (0.053, 0.109)**	—	**0.086 (0.051, 0.122)**	**0.081 (0.044, 0.118)**

*Model summary*			*F*(2,500) = 19.29, *P* < 0.001, *R* ^2^ = 0.08			*F*(4,498) = 8.98, *P* < 0.001, *R* ^2^ = 0.064
